# Biological ammonium transporters from the Amt/Mep/Rh superfamily: mechanism, energetics, and technical limitations

**DOI:** 10.1042/BSR20211209

**Published:** 2024-01-12

**Authors:** Gordon Williamson, Adriana Bizior, Thomas Harris, Leighton Pritchard, Paul A. Hoskisson, Arnaud Javelle

**Affiliations:** Strathclyde Institute of Pharmacy and Biomedical Sciences, University of Strathclyde, Glasgow, G4 0RE, U.K.

**Keywords:** Ammonium transporter, Amt/Mep/Rh, electrophysiology, energetics, methylamonium permease, rhesus protein

## Abstract

The exchange of ammonium across cellular membranes is a fundamental process in all domains of life and is facilitated by the ubiquitous Amt/Mep/Rh transporter superfamily. Remarkably, despite a high structural conservation in all domains of life, these proteins have gained various biological functions during evolution. It is tempting to hypothesise that the physiological functions gained by these proteins may be explained at least in part by differences in the energetics of their translocation mechanisms. Therefore, in this review, we will explore our current knowledge of energetics of the Amt/Mep/Rh family, discuss variations in observations between different organisms, and highlight some technical drawbacks which have hampered effects at mechanistic characterisation. Through the review, we aim to provide a comprehensive overview of current understanding of the mechanism of transport of this unique and extraordinary Amt/Mep/Rh superfamily of ammonium transporters.

## Introduction

Ammonium is a vital source of nitrogen for bacteria, fungi and plants, and a toxic metabolic waste for animals. Hence, ammonium transport across biological membranes is a process of fundamental importance in all living organisms. In 1994, the first genes encoding ammonium transporters were identified in *Saccharomyces cerevisiae* (mep1 for methylammonium permease) [1]. Two other Mep proteins (Mep 2 and 3) were later identified [2]. A parallel study identified and cloned Amt1 from the small flowering plant *Arabidopsis thaliana* by the expression of cDNA library in *S. cerevisiae* triple Δ*mep* mutants that could not grow on medium containing ammonium as sole nitrogen source [3]. Later, it was shown that the rhesus protein (Rh) is an Amt ortholog in vertebrates and that yeast *mep* mutants could be complemented functionally with the human Rh glycoprotein which can therefore act as an ammonium transporter [4,5]. Since then, members of the Amt/Mep/Rh protein family have been identified in almost all sequenced organisms; they constitute a unique and highly specific family of ammonium transporters [6,7].

### EcAmtB structure

**Figure 1:**
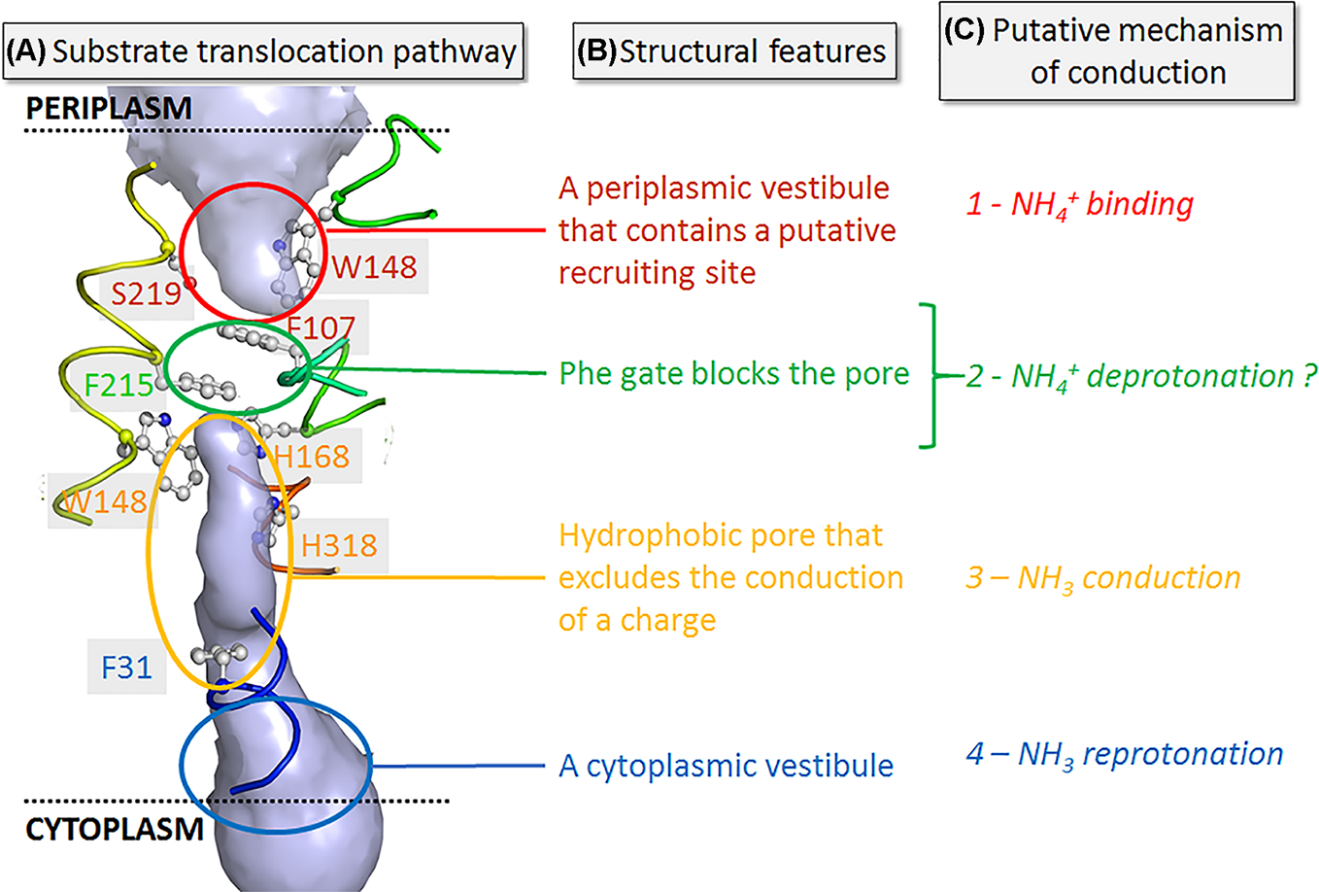
Substrate translocation path of AmtB (**A**) The pore region of one monomer is shown with the water-accessible volume represented in space filling representation (light-blue) (adapted from [16]). (**B**) Four mechanistically distinct segments: (i) periplasmic ammonium binding site, (ii) phenylalanine gate, (iii) central pore with twin-His arrangement and (iv) cytoplasmic vestibule can be discriminated. Selected, highly conserved residues are shown in ball-and-stick representation for the ammonium binding site (red), phenylalanine gate (green), central pore (yellow) and cytoplasmic vestibule (blue) where parts of the two transmembrane helices TM5 (His168) and TM10 (His318) are also shown. (**C**) Location and mechanism of the four steps in the putative translocation mechanism deduce from the structure.

To date, the *Escherichia coli* AmtB is the paradigmatic, most intensely studied member of the Amt/Mep/Rh superfamily of transporters with more than 20 high resolution structures reported in the Protein Data Bank (PDB) [8]. Structurally, AmtB forms homotrimers. At the centre of each monomer, is a potential periplasmic NH_4_^+^ binding site, delineated by the residues S219, W148, F107 and D160, followed by a hydrophobic pore (Figure 1). The binding site is separated from the pore by the partly stacked phenyl rings of residues F107 and F215, termed the ‘Phe-gate’. The high conservation of this ‘dynamic gate’ throughout the Amt/Mep/Rh family hints at an important mechanistic role, which is presently not understood. The side chains of two highly conserved histidine residues (H168 and H318 in AmtB), protrude into the lumen forming the so-called ‘twin-His’ motif (Figure 1). The strong hydrophobic nature of the pore suggests a high energy barrier for the conduction of NH_4_^+^. Therefore, the tentative consensus on the overall transport mechanism was that NH_4_^+^ binds to the transporter and gets deprotonated to NH_3_ before being translocated into the hydrophobic lumen of the channel [9–12].

High resolution structures have been solved during the last two decade for various Amt/Rh proteins. In a recent review, we compared all the known structures of prokaryotic Amts [8]; hence, in the context of the present review, we will compare the structure of AmtB with the structure of eukaryotic Mep and Rh protein.

### Comparison of ScMep2 and CaMep2 structure

**Figure 2:**
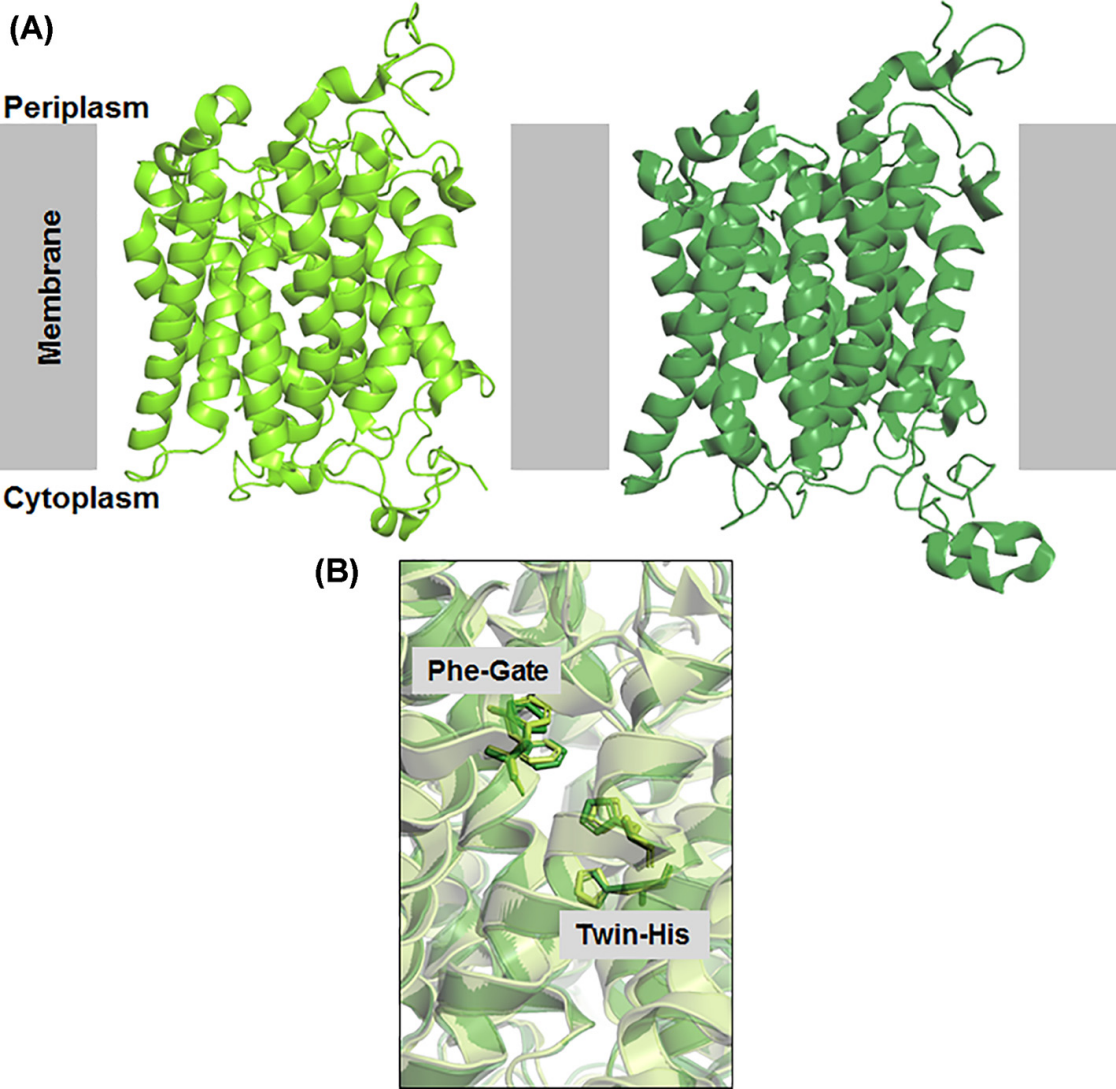
Comparison of fungal Mep2 (**A**) Cartoon models of X-ray crystal structures of Mep2 transceptors viewed from the side for *S. cerevisiae* Mep2 (light green) and *C. albicans* Mep2 (dark green). (**B**) Overlay of translocation pathway for ScMep2 (light green) and CaMep2 (dark green), showing the conservation of the Phe-gate and twin-His motif.

In 2016, two separate crystal structures of fungal Mep proteins, Mep 2 from *S. cerevisiae* and Mep2 from *Candida albicans*, were reported simultaneously [13]. Compared with each other, these proteins were highly similar (root mean square deviation of 0.7 Å) and thus will be discussed together (Figure 2). The crystal structures revealed the general architecture of the fungal Mep proteins to be highly similar to their prokaryotic counterparts: conserving archetypal trimeric organisation and pseudo-two-fold similarity across the 11TM helices in the monomers. In addition, the key structural features of the translocation pathway (S1 binding site, Phe-gate, twin-His motif) are all conserved (Figure 2).

### Comparison of CaMep2 and EcAmtB structure

**Figure 3:**
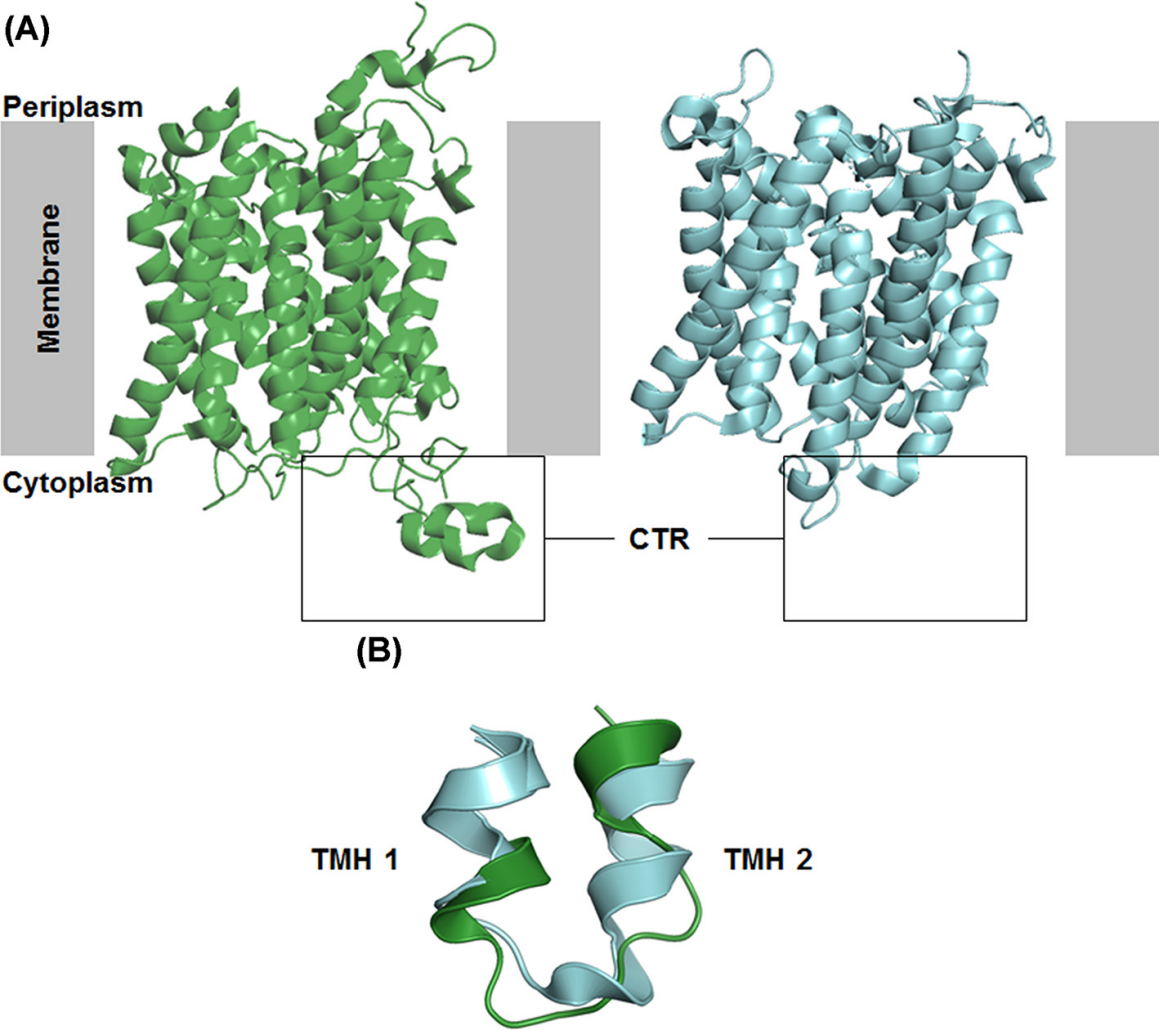
Comparison of *C. albicans* Mep2 and *E. coli* AmtB (**A**) Cartoon models of X-ray crystal structures of Mep2 transceptors viewed from the side for *C. albicans* Mep2 (dark green) and *E. coli* AmtB (cyan) The extended CTR has been boxed for comparison. (**B**) ICL1 in EcAmtB (cyan) and CaMep2 (dark green), showing unwinding and inward position of the fungal protein.

Significant deviation between Mep2 and previous bacterial structures was noted at 3 regions and could signify potential functional divergence. The first of these divergent regions is the N-terminus, which is extended by 20-25 resides compared with bacterial Amt. This causes the N-terminus to contact the ECL5 (Extracellular Loop 5) of the neighbouring monomer, substantially widening the extracellular domain and creating a more distinct binding site (Figure 3). The authors proposed that these changes would increase the stability of the Mep2 trimer however, as mutants lacking the N-terminus tail grew as well as WT on minimal media, they could not verify a functional role for the N-terminus. The Mep2 structures also differ from other ammonium transporters at the cytoplasmic exit of the translocation channel. In Mep2, the cytoplasmic end of TM2 is unwound, creating an extended intracellular loop 1 (ICL) that is shifted inwards compared to its bacterial counterparts (Figure 3). This alters the positioning of several residues, and ultimately results in a hydrogen bond interaction between Y49 (located at the C-terminus of TM1) and H342 of the twin-His motif which is not present in prokaryotic Amt. In addition, the ICL which links TM1-5 and TM6-10 is shifted by ∼10 Å, blocking the channel on the cytoplasmic side.

### Crystal structure of human RhCG

**Figure 4:**
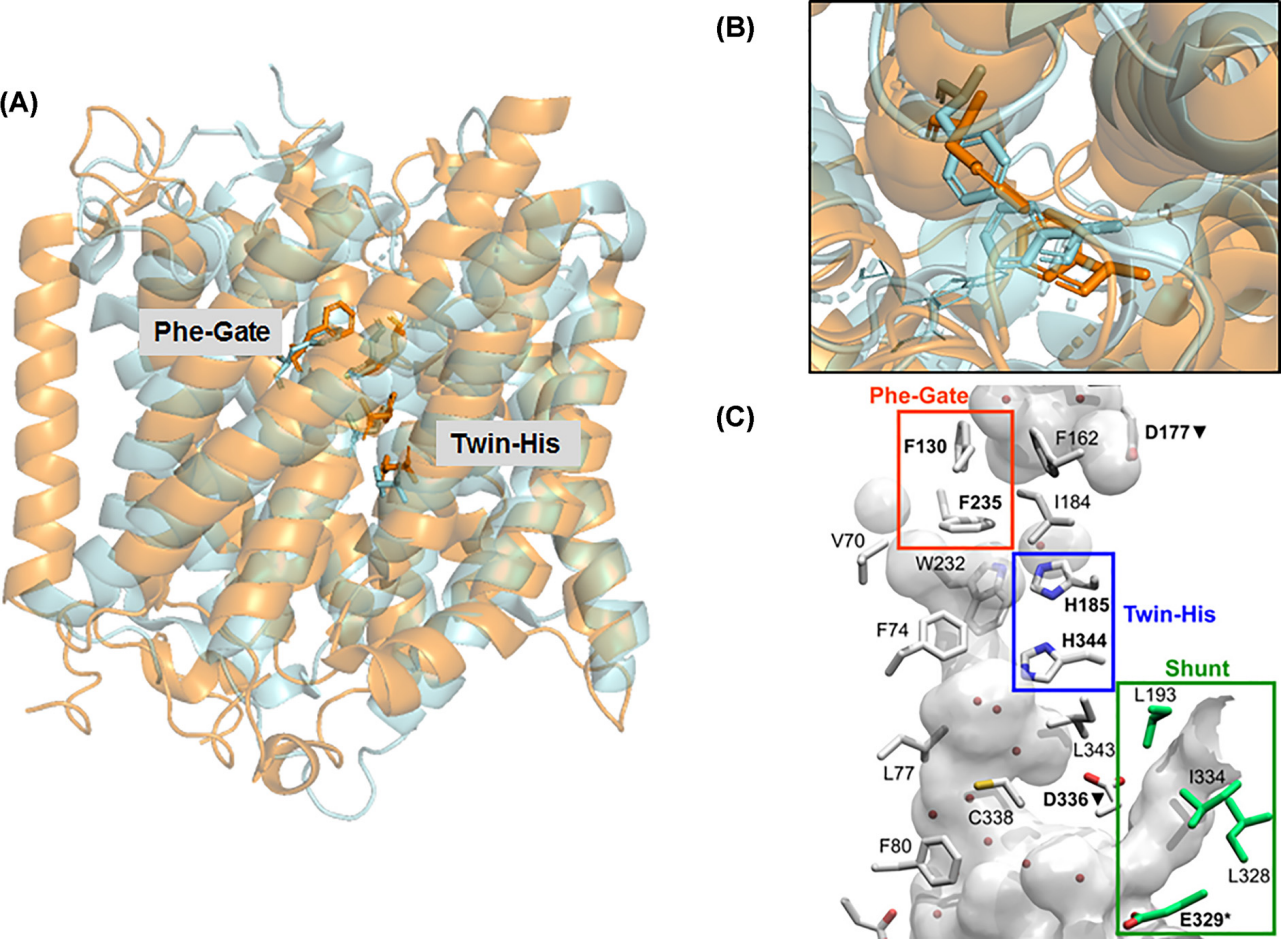
Comparison of AmtB and RhCG Overlay of a single monomer of AmtB (cyan) or RhCG (orange) as inserted into the membrane (**A**) Residues of the Phe-gate, and twin-His motif are displayed in the same colour as their respective monomer. (**B**) Comparison of the Phe-gate as viewed from the periplasm. (**C**) Side-view of the RhCG channel and shunt, as presented in [14].

In 2010, the crystal structure of RhCG – found in epithelial cells of the renal collecting ducts – was resolved at 2.1 Å [14]. This demonstrated that human Rh also adhered to a trimeric organisation and confirmed conservation of this conformation across the Amt/Mep/Rh superfamily (Figure 4). RhCG retains the pseudo-two-fold symmetry between TMH1-5 and TMH6-10 seen in bacterial Amt and Rh proteins, but also features a 12th N-terminal TMH. This helix, termed TM0, lies at the subunit interface of the trimer and varies in length across the different Rh isoforms, suggesting functional differentiation. As with the bacterial NeRh50, no S1 binding site was observed in the structure of RhCG. However, the authors suggested that acidic residues within the extracellular vestibule (E166, D218, D278, and E329) could serve to recruit NH_4_^+^. The phenylalanines of the Phe-gate are conserved, but in RhCG the outer phenylalanine (F130) does not obstruct the pore, resulting in an ‘open’ conformation with no barrier between the vestibule and the hydrophobic central pore (Figure 4). As with previously discussed members of the family, the twin-His motif is conserved and protrudes into the centre of this pore. Interestingly, Rh proteins share a common feature not seen in Amt or Mep proteins: a ‘shunt’ on the cytoplasmic face of the proteins (Figure 4). Whilst the function, if any, of the ‘shunt’ is unclear, the authors hypothesised that it may represent an alternative path for NH_4_^+^ entry, and NH_3_ delivery into the hydrophobic portion of the pore.

**Figure 5:**
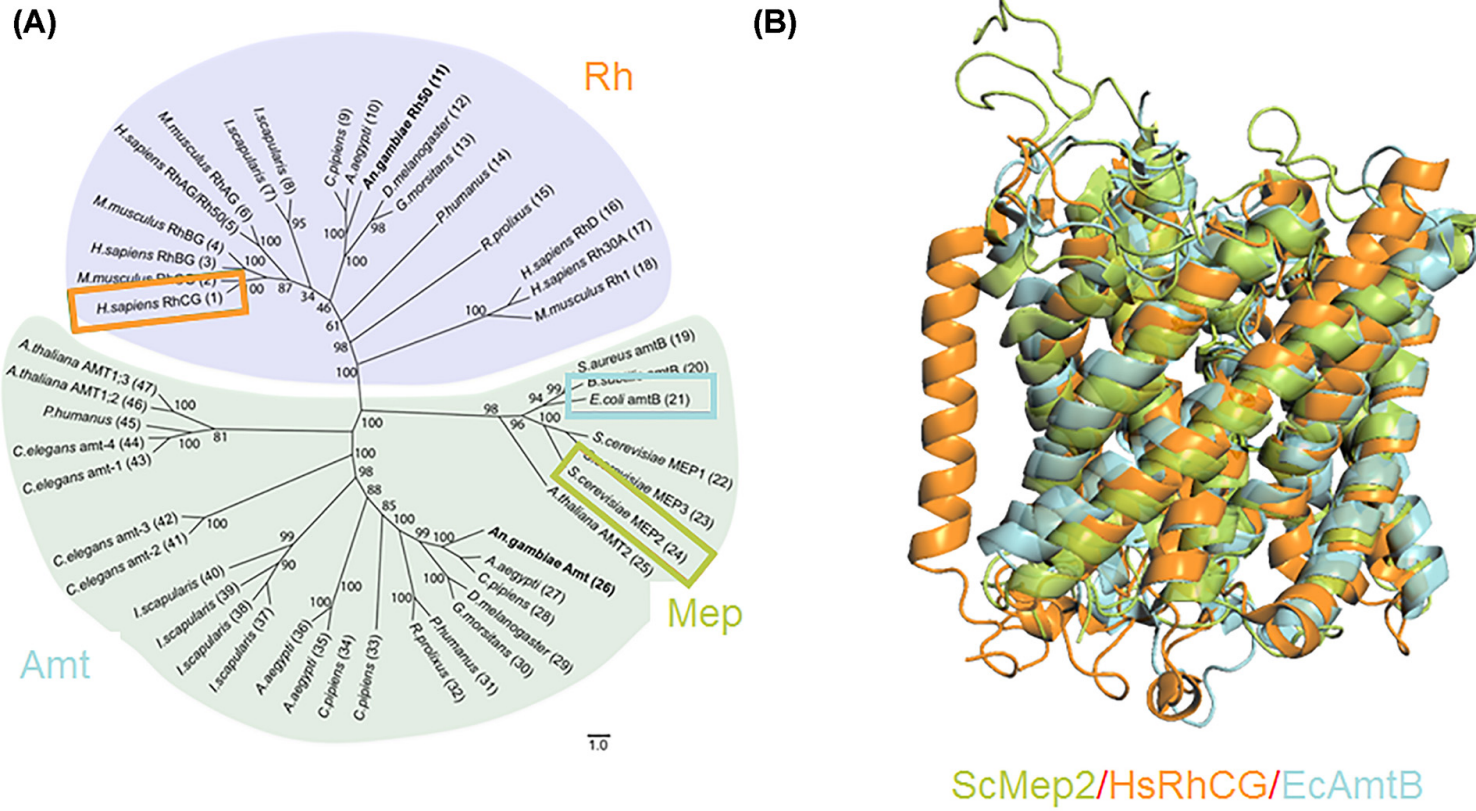
The Amt/Mep/Rh family is ubiquitous and highly structurally conserved (**A**) Neighbour-joining tree comparing ammonium transporters. Rhesus and Amt separate into distinct clades, whilst Mep appears within the Amt grouping. One example each of *E. coli* Amt (blue), *S. cerevisiae* Mep2 (orange), and *H. sapiens* RhCG (green) have been highlighted. (adapted from [54]) Scale bars are 0.5% and 1% corrected distance. (**B**) Structural Conservation of Amt/Mep/Rh protein. A monomer from *E. coli* Amt (blue), *S. cerevisiae* Mep2 (orange), and *H. sapiens* RhCG (green) aligned as inserted in the membrane. To highlight the conservation of the translocation pathway, the proteins have been oriented with the N-terminus at the top and C-terminus at the bottom.

Remarkably, the ubiquitous Amt/Mep/Rh family have strictly maintained their overall structure (Figure 5), despite individual members sharing low amino acid sequence identity.

### Highly conserved structure but functional diversification

**Figure 6:**
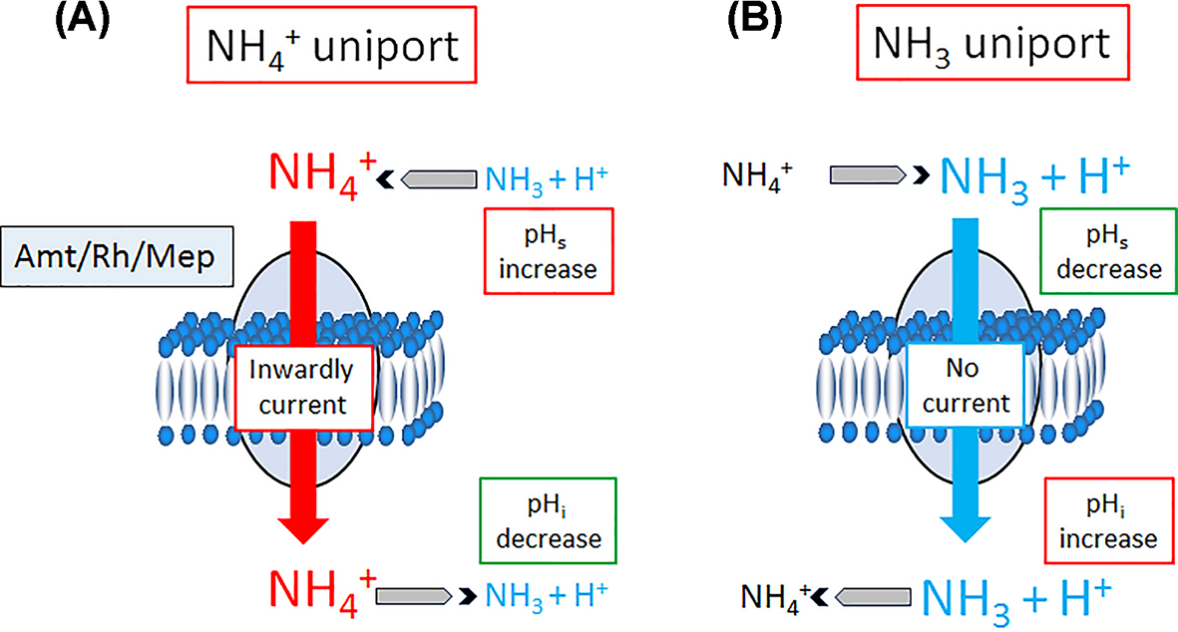
Influence on the current, surface pH (pH_s_) and internal pH (pH_i_) in oocyte as a function of the type of transport mechanism (**A**) If an Amt/Rh/Mep protein act as an NH_4_^+^ uniporter, it is expected to be associated with an inwardly orientated current and increase of the pH at the external surface (pH_s_) of the membrane and a decrease of the pH inside the compartment (pH_i_). (**B**) If an Amt/Rh/Mep protein act as an NH_3_ uniporter, it is expected that the translocation is electroneutral, and associated with a decrease of the pH at the external surface (pH_s_) of the membrane and an increase of the pH inside the compartment (pH_i_).

Despite sharing a common structure, the Amt/Mep/Rh proteins have become involved in a diverse range of physiological process spanning all domains of life, with new reports describing their involvement in biological processes published regularly, including selective ammonium transport across biological membranes, the mitigation of ammonia toxicity (due to nitrogen metabolic waste or environmental NH_3_), osmoregulation, maintenance of the physiological acid–base balance, ammonia detection in sensory structures (for organism development, finding a mate, a host, or food), and enhancing sperm survival and overall male fertility [8,15]. It is tempting to hypothesise that the different physiological functions gained by these proteins may be explained, at least in part, by differences in the energetics of their translocation mechanisms. Indeed, the energetics of ammonium transport through biological membrane can be very different. In solution, ammonium is in equilibrium between NH_3_ and NH_4_^+^ with an acid dissolution constant of 9.28, which means that a physiological pH, more than 99% of ammonium is present in its charged form. Because NH_3_ is uncharged, it can diffuse freely through the membrane bilayer down its chemical gradient, independently of the membrane potential. Following diffusion and because it is a strong base, NH_3_ triggers alkalinisation of the compartment it enters (Figure 6). In contrast, NH_4_^+^ is a weak acid, so the compartment is acidified as it accumulates (Figure 6). Additionally, NH_4_^+^ cannot easily diffuse through the membrane, due to its charge. As a result, the translocation of NH_4_^+^ through biological membranes is highly dependent on the membrane potential.

While structural and mechanistic understanding of the superfamily has expanded greatly over the last few decades [8], the energetic properties of Amt/Mep/Rh-mediated translocation remains poorly understood. Therefore, in this review, we will explore our current knowledge of energetics of the Amt/Mep/Rh family, discuss variations in observations between different organisms, and highlight some technical drawbacks which have hampered effects at mechanistic characterisation.

## Is transport active or passive?

### *Before the identification of genes encoding* bona fide *ammonium transporters*

The classical textbook definition of passive versus active transport is that passive transport down an electrochemical gradient occurs spontaneously, either by simple diffusion through the lipid bilayer or by facilitated diffusion through channels and passive carriers. In contrast, active transport occurs against the electrochemical gradient, and thus energy is required to empower transporter proteins to pump the solute against its electrochemical gradient. The first step to understand the energetics of Amt, Mep, and Rh proteins is to ascertain whether they act as passive or active translocator. In other words, do the transporters passively equilibrate the substrate alongside the existing chemical gradient, or do they have to actively overcome the electrochemical gradient? Classical approaches to discriminate between passive or energy-dependent transport mechanism focus on studying the kinetics of substrate accumulation in cells, cell-derived vesicles, patch-clamped oocytes, or proteoliposomes [16,17].

Early studies using these approaches, developed assays to measure ammonium accumulation in whole-cell samples of Gram-negative, Gram-positive bacteria, cyanobacteria and ectomycorrhizal fungi [17–20]. This is a complicated process for ammonium due to its high flux requirements for biosynthesis. However, the consensus view was that in these organisms they can accumulate ammonium inside the cell at concentrations 60-3000 times greater than the extracellular concentration [17]. This clearly indicated an active transport process as such ratios could not be achieved by passive diffusion (see [17] for review). At the time of these studies, the genes encoding these ammonium transporters had not been identified, which limited more detailed characterisation. These first genes encoding *bona fide* ammonium transporters would be identified 10 years later in 1994 [1,3]. In the years following this discovery, the superfamily of Amt/Mep/Rh transporters have been identified in all domains of life [6,8].

### *After the identification of genes encoding* bona fide *ammonium transporters*

This marked a paradigm shift in our understanding of the mechanism of ammonium transport. Instead of complying with the previous view that biological ammonium transporters actively translocate their substrate, a series of studies by Sydney Kustu speculated that instead Amt and Mep increased the rate of equilibration of uncharged ammonia across the cytoplasmic membrane using metabolic coupling with the glutamine synthetase in bacteria or acid trapping in the vacuole in yeast [21,22]. The authors also proposed that Amt and Mep may translocate substrate bidirectionally [23].

A passive transport mechanism was further substantiated when the first structure of the *Escherichia coli* ammonium transporter AmtB was published, revealing (i) a highly hydrophobic pore that would not accommodate NH_4_^+^ and (ii) the transporter structure was essentially unchanged when crystallised with or without the substrate, supporting the hypothesis of a channel with passive-like activity as opposed to active transport which is often associated with large conformational change during the transport cycle [9–11,16,24]. In addition, Khademi et al. [9], measured alkalinisation inside AmtB-containing artificial liposomes after an ammonium pulse, suggesting passive diffusion of NH_3_ (Figure 4) [9]. This was further supported in 2009 when Musa-Aziz et al. [25], expressed the *E. coli* ammonium transporter AmtB in oocytes and monitored the intracellular pH (pH_i_) and extracellular surface pH (pH_s_) following an ammonium pulse. They observed that a 0.5- or 5-mM ammonium pulse was associated with a sharp fall of pH_s_. They concluded that this was due to the protons released near the membrane after NH_4_^+^ deprotonation followed by NH_3_ diffusion through AmtB (Figure 6) [25]. This should be associated with a drastic alkalinisation inside the oocyte due to the reprotonation of NH_3_ releasing a hydronium (OH^−^) ion (Figure 6), but paradoxically they measured a decrease of pH_i_. However, numerous experimental observations contradict this model of AmtB acting as an NH_3_ uniporter. First, in a systematic effort we failed to reproduce the experiments published by Khademi et al. [16]. Secondly, Westerhoff and coworkers, using a very sophisticated systems biology approach, calculated that passive transport of NH_3_ would not be sufficient to sustain *E. coli* growth [26,27]. Thermodynamically, intracellular [NH_4_^+^] would not be able to exceed extracellular [NH_4_^+^] whilst maintaining an inward nitrogen flux. Therefore, if transport were passive, it would be expected that in an ammonium limited environment with low pH, *E. coli* would not be unable to grow [26,27]. However, *E. coli* does grow and continues to use ammonium as a nitrogen source in these conditions. In fact, AmtB is exclusively expressed under nitrogen limited conditions, when passive conductance would be insufficient. So, their conclusion was that AmtB should actively transport NH_4_^+^ (either net NH_4_^+^ transport or NH_3_/H^+^ co-transport) [26,27]. This hypothesis was experimentally proven using Solid-Supported Membrane Electrophysiology (SSME) in an *in vitro* essay with the purified protein reconstituted into artificial liposomes with two Amts from *Archaeoglobus fulgidus* and *E. coli* AmtB [12,28,29]. Using this SSME essay, it was clearly shown that the transport was electrogenic, equivalent to a net transport of a NH_4_^+^ molecule. The flux of NH_4_^+^ was estimated to be between 30 and 300 NH_4_^+^ ions per second per trimer [28], placing it in the flux range of secondary active transporters and far below a flux of 10^6^–10^8^ molecules of substrate transported per second expected for a passive channel. By combining this assay with a series of AmtB variants, the mechanism was further dissected, revealing a unique two-lane pathway for electrogenic NH_4_^+^ transport in the archetypal member of the family, the transporters AmtB from *E. coli* [12,30]. The pathway underpins a mechanism by which charged H^+^ and neutral NH_3_ are carried separately across the membrane after NH_4_^+^ deprotonation. Hence there is now a consensus that microbial Amts are active transporter [8,12,31]. This raised the question of the energetics of the translocation mechanism.

## What is the energy that drives the transport?

Although there is a growing consensus that most AMT/Mep/Rh proteins are active transporters, it is unclear how transport is powered. In this section, we will discuss the current hypotheses and associated evidence for the energy underpinning transport in different systems.

### Ammonium transport energetics in bacteria and cyanobacteria

Three main hypotheses have been put forward: ATP as a primary metabolic source of energy, metabolic trapping, or the proton motive force (PMF).

The hypothesis that the energy is provided by ATP is the easiest to formally exclude. Firstly, ATP-driven carrier proteins importing substrate into bacterial cells depend on the activity of an associated periplasmic substrate binding protein. However, spheroplasts of *Clostridium pasteurianum*, *Anacystis nidulans* and *E. coli* [32,33] are still active in transporting the radioactive ammonium substrate analogue [^14^C]-methylamine (MeA). Secondly, the structure of bacterial Amts proteins show that they lack the nucleotide binding domain typical of ATP-driven transporters [9–11,24,34].

Metabolic trapping is an attractive hypothesis, but evidence in support of this mechanism is scarce. A metabolic coupling between the Glutamine synthetase (GS) and AmtB in *E. coli*, *S. typhimurium*, and the free-living cyanobacterium *Anabaena variabilis* have been proposed [21,35–37]. This system would allow cells to concentrate glutamine instead of ammonium, and thus mitigate the toxicity of the latter. However, the initial MeA uptake activities in *E. coli* wild-type cells and cells that do not express the GS is similar. Also, the initial uptake activity of MeA is not affected when the wild-type cells of *E. coli*, *A. variabilis* and the filamentous cyanobacteria *anabaena flos-aquae* are treated with the GS inhibitors l-methionine sulphone (MSF) or L-methionine-DL-sulphoximine (MSX) [35,36,38]. Hence, Amts transport activity does not only depend on metabolic trapping in bacteria and cyanobacteria.

An ion electrochemical gradient combines the membrane potential (Δψ)a nd the ion concentration gradient. These components can either work additively, to increase the driving force on the ion across the membrane or can conflict and lower the driving force. In the case of the proton motive force (PMF), the two components: the inwardly orientated proton gradient, and Δψ with the inside negative with respect to the outside may favour an ammonium/proton symport mechanism. An early series of experiments point toward the PMF driving transport. Kleiner and co-workers showed that in *C. pasteurianum* and *Klebsiella pneumoniae*, inhibition of the ATPase, which maintains the PMF, also inhibits the MeA transport [32,39]. The use of protonophore Carbonyl cyanide p-(tri-fluromethoxy)phenyl-hydrazone (FCCP) and carbonyl cyanide m-chlorophenyl hydrazone (CCCP) on intact cells of *E. coli* or *Corynebacterium glutamicum* also collapses the PMF as well as MeA uptake [40].

The PMF has two components: the proton gradient, and the membrane potential (Δψ). Δψ is the dominant PMF component [41] and numerous lines of evidence point toward Δψ being the specific driving force for ammonium translocation. Using our SSME *in vitro* assay [12], we were able to demonstrate that in the absence of ammonium, a proton pulse in liposomes containing purified AmtB did not trigger a discernible current. Additionally, in the presence of ammonium, an inward-orientated pH gradient did not increase AmtB activity. This indicated that the proton gradient is not required for the activity of *E. coli* AmtB [12]. These data further show that AmtB cannot act as an uncoupler, which raises the question of proton selectivity and the coupling between NH_3_ and H^+^ transfer. However, the driving force created by the 200 mM ammonium concentration gradient used in these experiments is much bigger than the imposed delta-pH (out pH = 5.0, inside pH = 8.0), therefore interpretation of these data should be cautious [12].

The generation of an artificially high membrane potential by addition of valinomycin to K^+^-loaded cells of *C. pasteurianum* increase the MeA uptake [32]. Addition of the protonophores CCCP or Methyltriphenylphosphonium (TPMP) selectively decreases both Δψ and MeA transport in the cyanobacteria *Anabaena cylindrica* [42]. Collapsing specifically Δψ using valinomycin and a K^+^ gradient inhibit MeA transport in *Azotobacter vinelandii* [43]. MeA uptake studies in *C. glutamicum* cells demonstrated a high steady-state accumulation in the cytoplasm in the absence of any significant pH gradient [40,44]. Based on these data, the authors deduced that the membrane potential represents the driving force for ammonium transport in bacteria [40,44]. Similar conclusions were reached for AmtB from *E. coli*, when Fong *et al*. [45], observed accumulation of MeA ion (MeA^+^) in response to the electrical potential across the membrane [45]. In *A. fulgidus*, the thermodynamics of the different possible transport mechanisms of Amt-1 using free energy calculations were studied [46]. They concluded that the import of ammonium is likely predominantly driven by the Δψ since the transfer free energy of ammonium is much more sensitive to this component of the PMF than to the pH gradient. Maeda and co-workers [27] conducted a tour de force of engineering and modelling of a complex network for the ammonium transport and assimilation network in *E. coli*, involving AmtB, the regulatory proteins GlnK and GlnB, and the central N-assimilating enzymes and incorporated the highly complex interactions in to the model. Their simulations clearly shows that the Δψ in whole cells is required for ammonium uptake [27].

In the filamentous cyanobacterium *Anabaena flos-aquae*, MeA transport is completely abolished in the presence of 2 µM of the protonophore FCCP [38]. This suggested that ammonium transport depends mainly on the membrane proton gradient. However, FCCP is known to have some pleiotropic effects on cellular metabolism, including complete dissipation of the Δψ or depletion of intracellular ATP pool, therefore any interpretation of these data should be cautious [47].

Taken together, all these studies seem to indicate that at least for prokaryotic Amts, the membrane potential Δψ is essential for the transport activity.

### Energetics in Eukaryotes

### Ammonium transport energetics in yeast

The Baker’s Yeast, *S. cerevisiae*, possesses acidic vacuoles, where the pH is maintained between 5.5 and 6.2, (approximately 0.8 to 1.5 pH units lower than the cytosol) by two key players: the V-ATPase and V-type H^+^-ATPase [48]. The MeA transport activity of *Δvph1* cells, which cannot assemble the V-ATPase, or *Δvma1* cells which do not encode for a catalytic subunit of the ATPase is completely inhibited [22]. This originally led to the conclusion that energetic ammonium transport by the Mep proteins depends on trapping in the acidic vacuole. However, later studies seem to indicate that this process is more complicated and have provided a body of evidence indicating that the Mep2-type ammonium transporter had a different mechanism of transport compared to Mep1 and Mep3-type. This first hint of this was the discovery that the optimum pH activity for Mep1 is pH 6 whereas it is pH 4 for Mep2 [49]. More importantly, functional characterisation in oocytes of the frog *Xenopus* revealed that Mep2 mediates electroneutral substrate translocation while Mep1 conducts electrogenic transport [49]. A pHluorin reporter assay showed that the *in vivo* transport activity of Mep2 and Mep1 affected yeast cytosolic pH differently. After an ammonium pulse, the cells expressing only Mep1 showed an transient drop of the cytosolic pH but this was less marked in cells expressing only Mep2 [49]. It seems therefore clear that the mechanism of Mep2-type transporter differs from the Mep1/3-type, suggesting that the energetics of the transport may also be different. This would also explain why Mep2 acts as a signal transducer in the pseudohyphal growth signalling cascade, but Mep1/3 do not [31,50]. Later studies have reinforced this view. The MeA transport activity of Mep2 and Mep1 have been measured in the presence or absence of 50 µM of the protonophore CCCP. In cells expressing Mep2 only, MeA uptake was reduced to near background levels while transport was only marginally impacted in the cells expressing only Mep1 [49]. This suggests that the energetics of Mep1 and Mep2 are indeed different. However, it has been shown that protonophores (including FCCP and CCCP) have pleiotropic effects in yeast. Indeed, these protonophores do not only collapse the proton electrochemical gradient in mitochondria and across the plasma membrane, but also trigger a cytosolic acidification [51]. Hence, the popular use of these ‘protonophores’ to equilibrate H^+^ concentration across membranes appears to be an unreliable procedure that may lead to false and confusing interpretation. This makes it difficult to conclude with confidence that Mep2 depends on the proton gradient and Mep1 does not.

#### Ammonium transport energetics in ectomycorrhizal fungi

The ectomycorrhizal fungi *Paxillus involutus* and *Hebeloma cylindosporum* encode for three Amts transporters [18,20,52]. In *P. involutus*, the ammonium and MeA transport activity have an optimum pH between 4 and 5.5 [18]. The uptake systems of ectomycorrhizal fungi therefore seem to be adapted to the acidic pH of forest soil. To determine the energetic nature of ammonium translocation in these species, MeA transport activity has been measured in the presence of various metabolic inhibitors [18]. The protonophores CCCP and 2,4-dinitrophenol (DNP) strongly inhibit MeA uptake and NaN_3_, which reduces the internal pH by 1 or 2 units, results in moderate inhibition. These results indicate that MeA transport in *P. involutus* depends on the electrochemical proton gradient. The GS inhibitor MSX had no effect on the uptake of MeA, which suggests that metabolic trapping by the GS is not necessary for the transport activity. Addition of diethyl pyrocarbonate (DEPC), a histidine-specific alkylating reagent that modifies the histidine residues within proteins strongly inhibited MeA uptake, suggesting that a histidine residue would be instrumental in the translocation cycle. This was validated by later work which showed that the highly conserved ‘twin-His motif’, present at the centre Amt/Mep/Rh50 pore, is crucial for uptake activity [12,24,31].

Rotenone, SHAM and KCN, respectively, inhibitors of Complex I, alternative pathway and Complex IV of the mitochondrial respiratory chain, did not affect MeA uptake, nor did the mitochondrial ATPase inhibitor, arsenate, and glycolysis inhibitor, NaF, which indicates that the transport system was not directly under metabolic control. Gramicidin, a Na^+^/K^+^ ionophore, did not affect the transport of MeA, indicating that these cations did not take part in the uptake activity [18].

Take together these results indicate the PMF plays a role in ammonium transport in fungi, like in bacteria, but it is unclear if the Δψ or proton gradient is the dominant factor.

#### Ammonium transport energetics in insects

Insects detect their hosts and/or food by using ammonium olfactory detection to sense ammounium present in animal/human breath and sweat or decaying organic matter [53]. As part of this sensory apparatus, numerous insects express both Amt and Rh proteins in the same cell {Follstaedt, 2003 #199;Durant, 2019 #6257;Durant, 2020 #6258;Pitts, 2014 #6229}. The malaria vector mosquito, *Anopheles gambiae* encode for two ammonium transporters belonging to the Amt and Rh subfamily, AgAmt *and* AgRh50 (*AgRh50* encodes two spliced transcripts, *AgRh50a* and *AgRh50b*) [54]. The three proteins were expressed in oocytes and current were monitored in response to ammonium or MeA pulse ranging from 0.5 to 0.2 mM. Although all three proteins were able to restore growth to a transport deficient *S. cerevisiae* strain, only AgAmt produced a membrame potential dependant current in response to an ammonium pulse [54]. These results indicates that AgAmt acts as an electrogenic NH_4_^+^ transporter, while the two AgRh are electroneutral symporter or antiporter.

#### Ammonium transport energetics in plants

In plants, four separate mechanisms, each with distinct energetic characteristics, have been proposed for ammonium transport (Table 1).

1. NH_4_^+^ uniport,
2. NH_4_^+^ deprotonation followed by NH_3_/H^+^ conduction,
3. NH_4_^+^/H^+^ symport,
4. NH_4_^+^ deprotonation followed by the conduction of NH_3_ only.

**Table 1:** Transport Mechanism and energetics in plant AMTs

Organism/Protein	Mechanism*	Possible energetic	Reference
Tomato/LeAMT1;1	1	pH gradient independent Δψ dependent	[55]
Tomato/LeAMT1;2	1	pH gradient independent Δψ dependent	[56]
Tomato/LeAMT1;1	1 or 2	Δψ dependent	[57]
Arabidopsis/AtAMT1;1	1	pH gradient independent Δψ dependent	[58]
Tomato/LeAMT1;2	1 or 2	pH gradient independent Δψ dependent	[59]
Arabidopsis/AtAMT1;2	1 or 2	Δψ dependent	[60]
Arabidopsis/AtAMT1;1	1	pH gradient independent Δψ dependent	[61]
Wheat/TaAMT1;1	2	Proton gradients stimulate. Δψ independent	[62]
Lotus/LjAMT2;2	4	Proton gradients stimulate. Δψ independent	[63]
Arabidopsis/AMT2	4	pH gradient independent Δψ independent	[64]
Bean PvAMT1;1	3	Proton gradients stimulate. Δψ dependent	[65]
Arabidopsis/AtAMT1;2	2	Not determined	[66]
Rice/OsAMT1;1	1	pH gradient independent Δψ dependent	[67]
Liverwort/MpAMT1;2	2	pH gradient dependent Δψ dependent	[68]
Arabidopsis/AtAMT1;1, 1;2, 1;3 and 2;1	2 or 4	Not determined	[69]

* Mechanism: 1- NH_4_^+^ uniport, 2- NH_4_^+^ deprotonation followed by NH_3_/H^+^ conduction, 3- NH_4_^+^/H^+^ symport, 4- NH_4_^+^ deprotonation followed by the conduction of NH_3_ only.

The first characterisation of the transport cycle for a plant AMT was carried out on the tomato transporters LeAMT1;1 and LeAMT1;2 [56,70]. These tomato transporters were expressed in *Xenopus* oocytes and characterised using electrophysiological assays. For both proteins, ammonium pulses elicited currents that depended on the membrane potential but not the proton gradient. The *K*_m_ of both transporters varied with the applied voltage: as the voltage become more negative, the *K*_m_ measured for NH_4_^+^ decreased. From this, the authors concluded that LeAMT1;1 and LeAMT1;2 specifically bind NH_4_^+^ and mediate its potential-driven translocation (Table 1). This view was supported by later work which used a 3D model based on the structure of the *E. coli* AmtB combined with mutagenesis studies and revealed a functional ammonium binding site in the external vestibule of LeAMT1;1 [57].

In 2006, characterisation of another plant Amt, AtAMT1;1 from *Arabidopsis thaliana*, was reported [58]. The authors demonstrated that AtAMT1;1 can restore growth in *Δmep S. cerevisiae* (which are incapable of sustaining growth with ammonium as a sole nitrogen source) and that AtAMT1;1 activity in yeast is not pH-dependant. Additionally, they recorded an ammonium dependent current in oocytes expressing AtAMT1;1, indicating that the transport is electrogenic. By combining these data, the authors concluded that AtAMT1;1, like LeAMT1;1, acted as a membrane-potential-dependent NH_4_^+^ uniporter [58].

However, it is important to point out that for both AtAMT1;1 and LeAMT1;1, the data presented does not differentiate between mechanism 1 (NH_4_^+^ uniport) and mechanism 2 (NH_3_/H^+^ translocation following NH_4_^+^ deprotonation). In fact, in a later study based on electrophysiological analysis of tomato LeAMT1;1 and LeAMT1;2 expressed in oocyte, the authors recognized that they can't differentiate between the mechanism 1 and 2 (Table 1) [57,71]. In 2018, Ariz *et al*. [69], developed a detailed approach to explore this question. By using the natural chemical-physical properties of the N-isotopic signature linked to NH_4_^+^/NH_3_ interconversion, Ariz *et al*. [69] showed that *S. cerevisiae* cells expressing AtAMT1:2, 1:2, 1:3 and AtAmt2:1 were depleted in ^15^N relative to ^14^N when compared with the external ammonium source [69]. They show that this isotope fractionation can only be explained by the deprotonation of NH_4_^+^ before the translocation of NH_3._ However, the authors were unable to assess whether after deprotonation, the proton was translocated to the cytoplasm (mechanism 2) or recycled back to the periplasm (mechanism 4). By combining the deprotonation [69] with the electrogenicity measured in oocytes expressing plants Amt (Table 1 and references herein) it seems likely that AtAMT1;1, 1:2, 1:3, and LeAMT1:1, utilise mechanism 2. In contrast, the electroneutrality associated with the deprotonation favours the mechanism 4 for Lotus and Arabidopsis AMT2 activities (Table 1 and references herein).

Remarkably, the puzzle of plant AMTs mechanism of transport was not over. In 2014, a studies on Bean PvAMT1;1 propose that the transporter act as a NH_4_^+^/H^+^ symporter (Mechanism 3, Table 1 [65]). The authors heterologously expressed the transporter in oocytes and three main lines of evidence indicates the symporter activity in oocyte expressing PvAMT1;1, firstly, submillimolar ammonium and pH pulse triggered an inward currents, secondly, the amplitude of the current was proportional to the membrne voltage aply, finally, the ammonium pulse was assosciated the acidification of the oocyte cytoplasm.

Taken together, these data suggest that, despite the variety of mechanisms reported, the importance of the proton gradient and/or membrane potential is a common feature of plant AMTs.

### Energetics of ammonium transport in Rh proteins

As the most diverged member of the family, Rh have been proposed to have distinct functions compare to Amt/Mep proteins. However, yeast complementation assays [5,72] and functional studies [73,74] proved that Rh proteins facilitate ammonium transport. The hydrophobic nature of the bacterial NeRh50 and human RhCG pore revealed by X-ray crystallographic, suggested transport of ammonia (NH_3_) [14,75] but electrogenic transport of NH_4_^+^ has been repeatedly noted (Tables 2, 3, 5 and 6).

**Table 2:** Transport mechanism and energetics in NeRh50 proteins*

Mechanism*	Supporting evidence	Possible energetics	References
5&	- Electroneutral transport pH dependent - Resistance to MeA toxicity	pH gradient dependent Δψ independent	[72,75,76]
6	- Presence of CO_2_ binding pocket in crystal structure	CO_2_ concentration gradient	[77]
5	- MD simulation computing potentials of mean force and permeabilities for NH_3_	NH_3_ chemical gradient	[78]
2	- Electroneutral transport in lipososmes - Independent of pH gradient	pH gradient independent Δψ independent	[12]

* Mechanism: 2 - NH_4_^+^ deprotonation following by NH_3_/H^+^ conduction, 5 - NH_3_ uniport, 6 - CO_2_ transport, &- the activity may be bidirectional activity.

**Table 3:** Transport mechanism and energetics in RhAG proteins*

Mechanism*	Supporting evidence	Possible energetics	References
7&	**Human RhAG** - Yeast resistance to MeA toxicity, optimum an acidic pH -Ammonium excretion essay	pH gradient dependent	[5,82]
7	**Human RhAG** - Electroneutral transport in oocyte - Stimulated by outwardly pH gradient.	pH gradient dependent Δψ independent	[83]
5	**Human RhAG** - Rapid alkalisation of RBC ghost cell after NH_4_^+^ pulse - low Arrhenius activation energy	NH_3_ chemical gradient	[84]
1	**Mouse RhAG** - Rapid acidification of oocyte pHi - Depolarization of the cell	Δψ dependent	[85]
8	**Human RhAG** - Acidification in HeLa cell after ammonium pulse - Alkalinisation after an MeA pulse	Not none	[74]
8	**Mouse RhAG** - Acidification in oocyte after ammonium pulse - Alkalinisation after an MeA pulse	Δψ dependent pH sensitive	[85]
5	**Human RhAG** - Oocyte pH_s_ fall after ammonium pulse on oocyte	Potentially Δψ independent or NH_4_^+^ electrochemical gradient	[25,86]
8	**Mouse RhAG** -Transport both NH_4_^+^/NH_3_ and MeA^+^/MeA but; -Electrogenic transport is favour for ammonium -Electroneutral transport is favour for MeA	Δψ dependent NH_3_ chemical gradient pH dependent	[87]
6	- Intracellular pH changes - Surface pH changes -Ammonia-CO_2_ competition experiments - Translational knockdowns of Rh proteins	CO_2_ concentration gradient	[86,88,89]

* Mechanism: 1 - NH_4_^+^ uniport, 3 - NH_4_^+^/H+ symport, 5 - NH_3_ uniport, 6 - CO_2_ transport, 7 - NH_4_^+^/H^+^ antiport, 8 - Transport of both NH_3_ and NH_4_^+^, &- the activity may be bidirectional activity.

In keeping with the classically enigmatic nature of Rh proteins no less than eight different mechanisms, each with different energetic characteristics have been hypothesised. In this section, each of the proposed mechanisms of ammonium transport via bacterial and animal Rh50 proteins will be reviewed.

1. NH_4_^+^ uniport
2. NH_4_^+^ deprotonation following by NH_3_/H^+^ conduction
3. NH_4_^+^/H+ symport
4. NH_4_^+^ deprotonation following by NH_3_ transport only
5. NH_3_ uniport
6. CO_2_ transport
7. NH_4_^+^/H^+^ antiport
8. Transport of both NH_3_ and NH_4_^+^

### Ammonium transport in *Nitrosomonas europaea* Rh50

The chemolithoautotroph *Nitrosomonas europaea* is an Ammonia Oxidizing Bacteria (AOB) and it gains all its energy from the oxidation of ammonia to nitrate. A general assumption was that ammonia (NH_3_) rather than ammonium (NH_4_^+^) is the substrate for ammonia oxidation in *Nitrosomonas* [79]. The *N. europaea* genome encodes a single gene (*rh50*) belonging to the Amt/Mep/Rh family of ammonium transporters [6]. When expressed in *S. cerevisiae*, it mediates pH-dependent and bidirectional MeA transport, indicating that either neutral NH_3_ is transported alone, or NH_4_^+^/H^+^ are exchanged [72,76]. Weidinger et al. [72] also demonstrated that the MeA transport activity of *N. europaea* positively correlated with transcriptional level of *rh50* [72]. Another study compared MeA influx in *N. europaea* wild-type and *rh50* KO strains and observed decreased uptake rates in mutant cells [76]. Interestingly, it appears that AOB have developed a survival strategy at the molecular level by transcriptional regulation: under starvation conditions (deprivation of NH_3_ and CO_2_), nearly 70% of *N. europaea* genes are down-regulated. The expression level of ammonia monooxygenase (AMO) and hydroxylamine oxidoreductase (HAO) are maintained, while genes related to oxidative stress and transcription of Rh50 are up-regulated [80]. This suggests that Rh50 might be involved in the survival strategy of *N. europaea*, where it can sustain prolonged starvation periods, while preparing for the ammonia uptake and oxidation when it becomes available [81]. The presence of a highly hydrophobic pore identify in the crystal structure of *Ne*Rh50, prevents charge translocation, suggesting further that NeRh50 might translocate NH_3_ and/or CO_2_ [75,77]. However, a subsequent molecular dynamics study, simulating spontaneous full permeation events of ammonia and carbon dioxide across Rh50 revealed that the protein increases NH_3_, but not CO_2_ permeability in its crystallographic conformation, without the requirement for a major conformational change, indicating it functions as a channel rather than a transporter [78]. It was concluded that NeRh50 was highly representative of all Rh proteins; therefore, it was widely assumed that human Rh proteins would also act as NH_3_ channels. In 2020 however, it was shown by *in vitro* SSME assay that NeRh50 supports electrogenic activity [12].

### Ammonium transport in RhAG proteins

In 2000, the Rhesus-associated glycoprotein (RhAG) found in erythrocytes and often used for blood typing, was identified as a distant relative of existing Amt/Mep ammonium transporters through sequence homology [4]. Soon after this RhBG and RhCG glycoproteins found in almost all other tissues were shown to be involved in ammonia transport and metabolism [90,91], but the understanding of their true substrate or mechanism was lacking.

In 2000, Marini et al. [5], observed that expression of human RhAG in *Δmep* yeast conferred resistance against MeA toxicity, which provided the first evidence that RhAG mediates bidirectional transport of MeA [5]. This observation was consistent with a study showing that RhAG might be involved in ammonium efflux [92]. Two years later, Westhoff and colleagues expressed RhAG in *Xenopus* oocytes and MeA uptake was found to be independent of the membrane potential but dependent on an outwardly orientated gradient of protons, suggesting electroneutral NH_4_^+^/H^+^ exchange [83]. Two years late, Westhoff et al. [82], extended and confirmed their conclusion by showing that the excretion of MeA through RhAG expressed in yeast was pH-dependent, being optimal at acidic pHs, which according to the NH_4_^+^/NH_3_ pKa of 9.25, suggest that RhAG functions is a bidirectional NH_4_^+^/H^+^ antiporter [82]. Ever since, many studies have tried to elucidate the mechanism of RhAG-mediated substrate translocation. Pierre Ripoche tackled the question with a different approach and measured the pH inside human red blood cell ghost from genetic variants with defects of RhAG expression using stopped flow spectrofluorometry [84]. They measured alkalinisation inside the ghost cells in response to ammonium pulses, indicating the fast entry of NH_3_ (Figure 6). The Arrhenius activation energy for the translocation process is low, therefore they concluded that RhAG act as a NH_3_ channel [84]. Therefore, the work of Westhoff [93] and Ripoche et al [84] offers two opposing lines of evidence relating to the substrates for RhAG and no clear way to determine which was correct. However, a year later it was proposed that RhAG was able to transport both NH_3_ and NH_4_^+^ [74]. In this study, the authors pulsed HeLa cell with ammonium and monitor the intracellular pH (pH_i_). They observe the classical biphasic response for a cell membrane permeable to both NH_3_ and NH_4_^+^. In phase one, there is an initial pH_i_ alkalinization due to rapid passive entry of NH_3_, which steals a proton from an intracellular water molecule, forming NH_4_^+^ and releasing a hydroxide (OH^−^) ion, increasing pH_i_. This is followed by phase two - wherein NH_4_^+^ slowly enters the cells and dissociates to form NH_3_ and a proton H^+^, which slowly acidifies the cell and mediates recovery of pH_i_ (Figure 6 and Table 4). They repeated the same experiment in HeLa cells expressing RhAG and observed that the amplitude of the pH_i_ variation during the two phases were greater when compared the HeLa cell not expressing RhAG. Using a mathematical model to adjust the permeabilities to fit the pH_i_ profiles, they found that RhAG expression resulted in a three-fold and a two-fold increase of the NH_4_^+^ and NH_3_ permeability respectively and concluded that RhAG was able to transport both NH_4_^+^ and NH_3_ [74].

**Table 4:** Influence on the current, surface pH (pH_s_) and internal pH (pH_i_) in cells as a function of the type of transport mechanism*

Mechanism	Current	pH_s_	pH_i_
NH_4_^+^ uniport	yes	Increase	Decrease
NH_3_ uniport	No current	Decrease	Increase
Transport both	yes	Increase first then recovers if NH_4_^+^ flux is higher than NH_3_	Decrease first then recovers if NH_4_^+^ flux is higher than NH_3_
		Decrease first then recovers if NH_3_ flux in higher than NH_4_^+^	Increase first then recovers if NH_3_ flux in higher than NH_4_^+^

* pH_s_: pH at the external surface of the membrane. pH_i_: pH inside the compartment

**Table 5:** Transport mechanism and energetics in RhBG proteins*

Mechanism*	Supporting evidence	Possible energetics	References
7	**Human RhBG** - Ammonium pulse is associated with current in oocyte, not MeA pulse -^14^[C]-MeA uptake increases by outwardly pH gradient. - NH_4_^+^ transport induce pH_i_ alkalinisation.	Δψ dependent	[94]
1	**Mouse RhBG** - ammonium pulse associated with current and induce a pH_i_ acidification	Δψ dependent	[95]
1 or 8	**Mouse RhBG** -ammonium pulse associated with current and decrease pH_i_ -MeA pulse associated with current and increase pH_i_	Δψ dependent NH_3_ chemical gradient	[96]
8	**Mouse RhBG** -electrogenic transport is pH dependent -electroneutral transport is pH-independent	Δψ dependent NH_3_ chemical gradient pH dependent	[97]
5	**Human RhBG** - Oocyte pH_s_ fall after ammonium pulse on oocyte	Potentially Δψ independent or NH_4_^+^ electrochemical gradient	[86]
8	**Mouse RhBG** -Transport both NH_4_^+^/NH_3_ and MeA^+^/MeA but; -Electrogenic transport is favour for ammonium -Electroneutral transport is favour for MeA	Δψ dependent NH_3_ chemical gradient pH dependent	[87]
5&	**Human RhBG** - ammonium pulse associated with alkalanisation of cell expressing RhBC - pH_i_ acidification when cell loaded with ammonium were pulse with ammornium-free buffer	NH_3_ chemical gradient	[73]
7	**Mouse RhBG** - Electroneutral transport of MeA in oocyte - Stimulated by outwardly pH gradient.	pH gradient dependent Δψ independent	[98]

* Mechanism: 1 - NH_4_^+^ uniport, 5 - NH_3_ uniport, 7 - NH_4_^+^/H^+^ antiport, 8 - Transport of both NH_3_ and NH_4_^+^, &- the activity may be bidirectional activity.

In 2009 and 2013, Musa-Aziz et al. [25,86] expressed human RhAG in Xenopus oocytes and measured that an ammonium pulse was associated with an acidification of the surface pH (pH_s_). They interpret this as due to the protons released outside near the membrane after NH_4_^+^ deprotonation following NH_3_ diffusion through RhAG (Figure 6 and Table 4) [25,86]. Later, Caner et al. [87] completed this work and measured pH_i_, pH_s_, and whole cell currents following an ammonium pulse using an ion-selective microelectrodes and two-electrode voltage clamp in oocyte expressing RhAG [87]. The objective was to elucidate if RhAG acts as a NH_4_^+^ (or MeA^+^) electrogenic uniport, NH_3_ (or MeA) electroneutral uniport, or translocate both NH_4_^+^ and NH_3_ (and MeA^+^/MeA). When they pulsed the oocyte expressing RhAG with ammonium, they observed a current, an increase in pH_s_, (contrary to the observations of Musa-Aziz and colleagues who observed a pH_s_ decrease [25,86]) and an increase in pH_i_, indicating that RhAG acts as an NH_4_^+^ uniporter. However, both pH_s_ and pH_i_ slowly recover, which shows that RhAG transports both NH_4_^+^ and NH_3_ but the flux is higher for NH_4_^+^ than NH_3_ (Figure 6 and Table 4). Interestingly, when they used MeA, a current was measured but the pH responses were inverted: first the pH_s_ decreases and then the pH_i_ increases before both begin to recover. Hence it seems that Rh protein expressed in *Xenopus* oocytes handle ammonium and MeA differently; the electrogenic flux of the charged species dominate the electroneutral diffusion of the uncharged species for ammonium and inversely for MeA [87].

### Ammonium transport in RhBG protein

In a first study aimed at elucidating the mechanism of human RhBG translocation, Uwe Ludewig [94] recorded the current induced by ammonium and MeA pulse in Xenopus oocytes expressing RhBG using a two-electrode voltage-clamp. In addition, MeA uptake and intracellular pH_i_ measurements were performed. The results showed that MeA translocation in *Xenopus* oocytes expressing RhBG is electroneutral and enhanced by increasing an outwardly orientated H^+^ gradient. Moreover, an ammonium pulse did not elicit a current but did result in internal alkalinisation. These results lead to the conclusion that human RhBG acts as an NH_4_^+^/H^+^ antiporter [94].

Studies on *Rhbg* knockout (KO) mice demonstrated lowered urinary ammonium excretion, while HCl-induced acidosis increased RhBG protein expression in healthy mice [99]. The transport mechanism of mouse RhBG and RhCG have been studied by electrophysiology in *Xenopus* oocytes. It was shown that oocytes expressing RhBG can transport MeA and that this activity is independent of the membrane potential. Moreover, the transport was dependent on an outward-directed pH gradient, suggesting an NH_4_^+^/H^+^ antiporter activity, in agreement with Uwe Ludewig’s data on human RhBG [94,98]. Interestingly, while adapting Uwe Ludewig's technique on mouse RhBG, Nakhoul et al. [95] reported that an ammonium pulse on oocytes expressing RhBG triggers a Δψ dependent current and a significant pH_i_ decrease. These data suggest, contrary to the conclusion drawn before by Uwe Ludewig, that RhBG acts as a strict NH_4_^+^ transporter [95].

Nakhoul and colleagues clarified this apparent discrepancy in a series of three papers published between 2010 and 2015 [87,96,97]. Firstly, in 2010 Nakhoul et al. used ion-selective microelectrodes and voltage-clamp experiments to measure currents and intracellular pH changes in whole cells after an ammonium or MeA pulse [96]. After an ammonium pulse, they measured a current and acidification inside the oocytes, consistent with their 2005 paper, which seemed to confirm that RhBG acts as an NH_4_^+^ uniporter (Figure 6) [95,96]. However, while a MeA pulse induced a current as ammonium did, it also induces alkalinisation of inside the oocyte instead of acidification. Therefore, they concluded that as for RhAG (see above), RhBG handles ammonium and MeA differently: NH_4_^+^ is transported electrogenically but MeA can either be transported electrogenically (as charged MeA^+^) or in an electroneutral manner (via diffusion of neutral MeA) [96]. The authors then expanded their experimental design to include surface pH measurements in addition to current and pH_i_ and probed the effect of pH on the activity of RhBG-expressing oocytes. The pH_i_ of the oocytes was acidified by 0.2–0.3 units using butyrate, while the extracellular pH (pH_o_) was adjusted to either 6.5, 7.5, or 8.2 prior to pulsing with substrate. Their data show that the electrogenic transport (of either NH_4_^+^ or MeA^+^) is pH sensitive: stimulated by alkaline pH_o_ but inhibited by acidic pH_i_ or pH_o_, however the electroneutral translocation of MeA is not affected by pH [97]. In a third paper Nakhoul *et al*., expanded their experimental design further: this time including an electrode to measure pH at the surface of RhBG-expressing oocytes (in addition to the previous measurements) [87]. These data demonstrate that RhBG can facilitate electroneutral transport of NH_3_ and thus the demarcation between electrogenic and electroneutral transport of MeA^+^/MeA in RhBG is also extended to ammonium. As with RhAG, RhBG handled the two substrates differently: the electrogenic flux of charged NH_4_^+^ dominated over the electroneutral diffusion of neutral NH_3_ while the inverse was true for MeA (Figure 6 and Table 4) [87]. From this, they deduced that the electroneutrality previously observed by Ludewig et al. [94], in RhBG-expressing *Xenopus* oocytes was due to the fact that they used MeA and not ammonium, hence the electrogenic transport of MeA^+^ was masked by the diffusion of MeA [94]. Surprisingly in 2013, Geyer and colleagues [86] observed an acidification of oocyte pH_s_ expressing RhBG, concluding to an NH_3_ uniport mechanism (Figure 6) [86].

A completely different approach was developed by Pierre Ripoche in Paris [73]. In this study, they measured the pH_i_ of HEK-293 (human embryonic kidney) and MDCK (Madin–Darby canine kidney) cells expressing RhBG or RhCG following an ammonium or MeA pulse. For both Rh proteins, an ammonium or MeA pulse triggers a pH_i_ alkalinisation; moreover, when the cells were loaded with ammonium and pulse with an ammonium-free buffer, they measured a pH_i_ acidification. This suggests clearly that RhBG and RhCG act as a bidirectional NH_3_ channel [73].

### 3-5-4-RhCG Protein

In a triple *mep*-deficient yeast strain, human RhCG was shown to function as a bidirectional NH_4_^+^ transporter [5]. Bakouh et al. [97] subsequently expressed RhCG in *Xenopus* oocytes and used voltage-clamp experiment at various external pHs to control both NH_4_^+^ and NH_3_ concentrations independently of each other. They measured an inwardly orientated current following an ammonium pulse, revealing electrogenic transport. This current was drastically increased when the external NH_4_^+^ concentration was kept constant while the external concentration of NH_3_ was increased 3-fold by increasing external pH by 0.5 unit. They concluded that RhCG induced NH_4_^+^ transport depending on [NH_3_] but could not identify the underlying mechanism [101]. In a later study, it was shown that RhCG expressed in *Xenopus* oocytes transport MeA independently of the membrane potential. Moreover, the transport was also dependent on an outward-directed pH gradient, suggesting an NH_4_^+^/H^+^ antiporter activity [98].

In 2006, Mayer and colleagues [71] expressed human RhCG in *Xenopus* oocytes and in yeast and combined electrophysiology, with MeA uptake assays, and pH_i_ measurements to characterise the protein activity. Their results show that RhCG transports both ammonium and MeA and that uptake was strongly favoured as extracellular pH increased. This clearly indicated that RhCG mediates electroneutral NH_3_ transport [71] (Table 6). This conclusion was further supported in 2013 when Geyer and colleagues [86] observed an acidification of *Xenopus* oocyte pH_s_ expressing RhCG following an ammonium pulse, also indicating an NH_3_ uniport-type mechanism (Figure 6) [86]. This was confirmed two years after, in *Xenopus* oocytes expressing RhCG, where Caner et al. [87] could not measure an inward current following a pulse of either ammonium or MeA, but they were able to measure a significant acidification of pH_s_ with ammonium but not with MeA [87] (Table 6). These data seem to indicate that RhCG transports NH_3_, but not MeA. Surprisingly however, NH_3_ transport by RhCG is not accompanied by alkalinisation in *Xenopus* oocytes (Figure 6). This is likely due to heterologous expression in oocytes results in ammonium being handled differently compared to other cells (see paragraph below ‘*Technical limitations studying ammonium transport’*). Nevertheless, the authors concluded that RhCG acts as an NH_3_ uniporter [87].

**Table 6:** Transport mechanism and energetics in RhCG proteins*

Mechanism*	Supporting evidence	Possible energetics	References
7&	**Human RhCG (formerly RhGK)** - Yeast resistance to MeA toxicity, -Ammonium excretion essay	pH gradient dependent	[100]
8	**Human RhCG** Electrogenic transport Current depend on [NH_3_]	Δψ dependent	[101]
5&	**Human RhCG** - ammonium pulse associated with alkalanisation of cell/lipososme/erythrocyte vesicles expressing RhBC - pH_i_ acidification when cell loaded with ammonium were pulse with ammornium-free buffer	NH_3_ chemical gradient	[73,102]
7	**Mouse RhCG** - Electroneutral transport of MeA in oocyte - Stimulated by outwardly pH gradient.	pH gradient dependent Δψ independent	[98]
5	**Human RhCG** -Electroneutral transport of ammonium and MeA in oocyte -Increase flux when increase external pH -MeA transport essay in yeast	NH_3_ chemical gradient	[71]
5	**Human RhCG** - Alkalinisation inside liposomes containing purified RhCG after an ammonium pulse	NH_3_ chemical gradient	[14]
5	**Human RhCG** - Oocyte pH_s_ fall after ammonium pulse on oocyte	Potentially Δψ NH_4_^+^ electrochemical gradient	[86]
5	**Mouse RhCG** -pHs decrease upon ammonium pulse, but not with MeA	NH_3_ chemical gradient	[87]
4	**Human RhCG** -Molecular and quantum mechanical calculations	Potentially Δψ independent or NH_4_^+^ electrochemical gradient	[103]

* Mechanism: 3 - NH_4_^+^/H+ symport, 4 - NH_4_^+^ deprotonation following by NH_3_ transport only, 5 - NH_3_ uniport, 7 - NH_4_^+^/H^+^ antiport, 8 - Transport of both NH_3_ and NH_4_^+^, &- the activity may be bidirectional activity.

Work using stopped-flow spectrophotometry to measure pH_i_ changes in HEK-293 cells expressing RhCG, erythrocyte vesicles and liposomes containing purified RhCG was found to show that ammonium and MeA pulses are associated with a pH_i_ decrease, indicating that RhCG translocates NH_3_ [73,102]. Measurements conducted at different temperatures revealed that the translocation mechanism exhibits a low Arrhenius activation energy, suggesting a channel-like rather than transporter-like mechanism for RhCG (Table 6) [73,102].

In 2010, the high-resolution structure of human RhCG was published alongside characterisation of the protein reconstituted into functional liposomes [14]. The authors measured an alkalinisation in the lumen following an ammonium pulse, and thus concluded that RhCG is an NH_3_ uniporter (Table 6) [14]. Notably, they used the same experimental design used six years prior to conclude that EcAmtB was also an NH_3_ uniporter [9]. However, a later systematic effort failed to reproduce these data [16]. Following the publication of the high-resolution structure of RhCG [14], a team lead by Simon Bernèche used molecular and quantum mechanical calculations to characterise the mechanism of RhCG translocation [103]. They showed that RhCG recruits NH_4_^+^ which is subsequently deprotonated via the ‘twin-His’ motif in the pore and the proton is transferred back to the extracellular vestibule through a hydrogen bond network, resulting in the net transport of NH_3_ [103].

## Rh proteins and CO_2_ transport

Analysis of evolutionary conservation and diversification suggested that Rh may have adapted to transport neutral CO_2_, in place of or in parallel to NH_3_, as a means of maintaining an appropriate pH homeostasis [6]. The first experimental report claiming Rh involvement in CO_2_ transport came from studying expression of the RH1 gene in the green alga *Chlamydomonas reinhardtii* [104]. The authors showed that expression of RH1 increased in cells grown in air supplemented with 3% CO_2_ and concluded that Rh1 and, by extension, Rh proteins are the long-sought gas channels for CO_2_ [104]. A subsequent study provided further evidence for this claim by showing that inhibiting expression of Rh reduces the response to extracellular CO_2_ in *C. reinhardtii* [105]. In another study, which used direct ammonia–CO_2_ competition experiments affected both ammonia and CO_2_ excretion in the Zebrafish, *Danio rerio*, suggesting that Rh proteins may serve as channels for both CO_2_ and NH_3_ [89]. Identification of a potential CO_2_ binding pocket in the *Ne*Rh50 crystal structure, further suggested that Rhesus proteins might act as gas channels for NH_3_ and/or CO_2_ [77]. These findings prompted the investigation of human Rhesus proteins and their involvement in CO_2_ transport [88,106]. Analysis of red blood cell ghost membrane vesicles deficient in RhAG expression and knockout mice displaying well-characterized protein defects, showed that even a small drop in the production of this protein significantly reduces CO_2_ exchange [88,106]. It was found that, RhAG accounts for ∼50% of CO_2_ transport [88]. This was also supported in another study showing that RhAG represents a channel for extra-renal transport of CO_2_ molecules based on changes in surface pH in *Xenopus* oocytes expressing RhAG and aquaporin AQP1 [107]. However, RhAG demonstrated four-fold greater preference for passage of NH_3_ versus CO_2_ leaving the results rather open ended.

At the same time, experimental characterisation NeRh50 *in vivo* provided no evidence of a CO_2_-dependent growth effect in a KO mutant [76]. Another argument against Rh involvement in CO_2_ transport was presented in a later report monitoring transmembrane CO_2_ flux by imposing a CO_2_ concentration gradient across planar lipid bilayers and detecting the resulting small pH shift in the immediate membrane vicinity. The study concluded that Rh protein facilitated transport of CO_2_ through biological membranes is highly improbable [108]. In addition, no putative CO_2_ binding pockets were detected in the crystal structure of human RhCG, which was proposed to be representative for all Rh homologues [14]. Finally, a recent study showed that during long equilibration molecular dynamics simulations the CO_2_ molecules do not show any tendency to diffuse across the periplasmic vestibule of either bacterial or human Rh50 protein [109].

## Technical limitations studying ammonium transport

A recurring theme through this review is that despite using the same, or similar techniques, various authors (ourselves included) have produced conflicting evidence regarding the mechanism of Amt/Mep/Rh transport. As such, the various methodologies used over the past four decades to explore the questions of specificity and mechanism of conduction of Amt/Mep/Rh protein require a reasoned critical appraisal. In this section, we want to look beyond the heated debates concerning the Amt/Mep/Rh transport mechanism and instead highlight some potential technical drawbacks. We believe that understanding the limitations of methodology, combined with the application of current and emerging approaches will fuel an expansion in our mechanistic understanding of this extraordinary superfamily of transporters.

### Choice of experimental system

#### *Xenopus laevis* oocytes

Amt/Mep/Rh proteins are present in a diverse range of organisms, and thus are placed in a variety of biological and physiological contexts. As a result, it is likely that Amt/Mep/Rh from different organisms fulfil different functions and thus differ mechanistically. Despite this, the majority of the studies are carried out *in vivo* using intact cells (typically *E. coli*, *S. cerevisiae*, *X. laevis* oocytes, or human cells) or cell-derived vesicles (red blood cell ghost, *E. coli* spheroplasts or vesicles). The parameters used as proxies for the detection and measurement of ammonium conduction (pH change, electric current, uptake of labelled analogue) are likely to be affected by other physiological phenomena and are thus difficult to control and interpret with confidence. The best example is the use of *X. laevis* oocytes to study biological ammonium transporters. As mentioned in the introduction, oocytes possess endogenous ammonium-activated channels and thus handle ammonium in an unusual manner compared with the native hosts of the tested transporters [110]. Detailed study of ammonium handling in *Xenopus* oocytes has revealed three keys ways in which they differ from other cells [25]. With the acido/basic equilibrium ${\text{NH}}_{4}^{+}⇆{\text{NH}}_{3}+{\text{H}}^{+}$ with a pKa of 9.25 in mind (see also Figure 6), first, after an ammonium pulse, the pH inside the oocyte falls, while in other cells passive diffusion of NH_3_ through the membrane is associated with intracellular alkalinisation (Figure 6). Second, abrupt removal of external ammonium immediately after an ammonium pulse leads to alkalinisation inside *Xenopus* oocytes. Normally, however, this should lead to acidification, as NH_3_ would be expected to diffuse out, displacing the NH_4_^+^/NH_3_ equilibrium towards the right, and yielding a proton [25]. Third, removal of external ammonium following a pulse would be expected to trigger a sharp increase of the pH at the surface of the oocyte due to the acid/basic NH_4_^+^/NH_3_ equilibrium being completely displaced to the left. However, the authors did not measure any significant pH_s_ variation [25]. In an attempt to explain this erratic behaviour of oocytes towards ammonium, the authors developed between five and seven different models which are too extensive to reproduce here. *Xenopus* oocytes seems to be particularly adept at avoiding ammonium-induced increases in pH_i_, most likely as an adaptation to protect the egg from toxic levels of ammonium arising from the decay of organic matter in pond water [25]. The poorly elucidated behaviour of ammonium handling in oocytes, combined with variations in the use of ammonium and/or methylammonium, specific parameter measured, and detection method may explain how the same basic approach has given rise to such various conclusions on the translocation mechanism of Rh proteins.

#### Heterologous expression in prokaryotes

Heterologous expression of Amt/Mep/Rh proteins in bacterial systems comes with its own limitations. The ^13^N radionuclide has a short half-life (∼10 min), so radiolabelled NH_4_^+^ is not viable for biological studies. As a result, [^14^C]-MeA uptake assays have long been used to assess Amt activity *in vivo* [111]. Bacterial glutamine synthetase (GS) is able to convert MeA into methylglutamine and thus introduce it into the larger cellular metabolism [112]. H ence in [^14^C]-MeA uptake assays, free intracellular MeA is removed upon washing the cell and consequently the radioactivity measured is only from metabolized products of MeA. As a result, kinetic parameters determined in this way do not relate to MeA translocation but rather are representative of its assimilation [113]. Hence, any kinetic data obtained from MeA uptake assays *in vivo* must be analysed and interpreted very carefully before drawing conclusions on the translocation mechanism [114]. Jayakumar and Barnes [113], followed by Javelle et al. [36], developed an assay that does not require a washing step. The benefit of this assay is that it allows determination of both free and metabolized intracellular MeA and (using a GS mutant) allows uncoupling of MeA uptake from subsequent assimilation [113,114]. However, the absence of a washing step also makes the assay extremely sensitive to pipetting error, hence can vary from experimenter to experimenter.

#### Artificial proteoliposomes

An *in vitro* assay set up with purified protein would not suffer from such problems and would also make it easier to vary experimental parameters like substrate concentration, pH, and presence of potential inhibitors. In particular, it allows assessment of the activity of variant proteins designed to test mechanistic hypotheses under controlled conditions. Such an assay has been developed by purifying and reconstituting Amt protein from *A. fulgidus* and *E. coli*, along with Rh50 from *N. europaea* and human RhCG into artificial liposomes [12,28,29,102]. The problem here is that it is impossible to control the exact quantity and the orientation of the protein inside the lipososmes. In these studies it was demonstrated that AfAmt was inserted 100% inside out (IO), while for EcAmtB and NeRh50 it was a mixture of IO and right side out (RSO). This complicates interpretation as measurements include activity resulting from both influx and eflux at the same time. Also, these assays use enormous ammonium concentration (5–200 mM), which creates a signfiicant driving force that might force mechanistic steps that would not occur at physiologically concentrations or mask steps that do occur physiologically. Another problem with these system is the lipid composition of the proteoliposomes. Electrophysiology measurements coupled with MD simulations revealed that phosohtidylglycerol is an essential cofactor for AmtB activity and, in its absence, AmtB cannot complete the full translocation cycle [115]. Another study further investigated the lipid selectivity of AmtB from *E. coli* in heterogeneous nanodiscs. Gas-phase ejection and solution-phase detergent ‘flash’ extraction analysis of AmtB revealed that the protein has a few tight binding sites for phosphtidylcholine, is selective for binding phosphtidylglycerol overall, and is nonselective for phosohtidylethanolamine [116]. Thus far, lipid-dependency has only been demonstrated for AmtB, but given the conserved structure of the family it is likely to be a universal feature for the family. Hence it is important to identify the optimum lipid composition for the specific ammonium transporter under investigation.

### X-ray crystallography and MD simulation

They were big expectations that high resolution structures would help to understand the mechanistic of these transporters. High resolution structures have been solved during the last decade for various Amt/Mep/Rh proteins (for review see [8] and reference herein). Unfortunately, the anticipated elucidation of the transport mechanisms from these 3D structures has not been met, as all the structures are very similar when generated in the presence or absence of ammonium and show the same inward-facing state of the protein. There are no significant differences in the crystal structures of Amt/Mep and Rh proteins that can clearly account for different mechanism of translocation. Consequently, all structural model based on authentic 3D structures will also all look similar and are largely uninformative. The incredible ‘structural rigidity’ has also complicated the application of MD simulations aimed at interrogating hypotheses on the mechanism of transport. Resulting in an aspect that has largely ignored – the dynamics associated with the translocation mechanism in these proteins. It is, therefore, essential to develop an alternative approach to protein crystallography to observe different conformations and obtain more structural information. In this context, developing a combined approach using the stabilisation of the protein in a native lipid environment combined with Cryo-electron microscopy and neutron/X-ray scattering may offer a way forward [115].

### Methylammonium is an imperfect substrate analogue

The use of ^14^[C]-MeA transport essay, as discussed above, has become an almost universal tool for assessing Amt activity *in vivo* in bacteria, yeast, *Xenopus* oocytes and fungi [1,8,18,94]. However, numerous observations raise clear concerns over the validity of MeA as a substrate analogue in any system (either *in vivo* or *in vitro*) to solve mechanistic questions in ammonium transporters. In some variant proteins, the activity measured with MeA as a substrate does not mirror the activity measured using ammonium [12,31,114,117,118]. This is particularly apparent in *Xenopus* oocytes, where it has become clear that Rh proteins handle ammonium and MeA differently [87,94–96]. It is possible that the degree of discrimination between ammonium and MeA differs between individual proteins: limiting potential comparisons between different transporters. Indeed, Amt/Mep/Rh proteins selectively transport a very small molecule (NH_4_^+^), adding a methyl group to that is a significant increase in size. This will affect how the speed and efficiency of pore entry and translocation. Additionally, the p*K*a of methylammonium is ∼10.6, such that the equilibrium of the substrate and the analogue when interacting with the protein are not the same, which can lead to problems during the translocation cycle.

### Activity measurements are incomplete

Different studies have measured different phenomena as a proxy for ammonium transport activity. The two most common approaches are to monitor for a change in pH (either inside the vesicle/oocyte or its surface) or measuring an electric current, but both introduce complications. Measuring only current can hide the diffusion of the neutral species as it has been the case for RhBG expressed in *Xenopus* oocytes [87,94] and is also problematic with electrophysiology on liposomes [28,29]. Conversely, measuring only pH_i_ variation may favour the detection of electroneutral over electrogenic components of the translocation mechanism (Table 4 and Figure 6). Another problem with pH_i_ measurements lies in the detection method: often a fluorescent dye or pH-sensitive electrodes. A disadvantage of conventional electrodes is that the impalement depth is hard to quantify and pH-sensitive electrodes may measure bulk cytosolic pH rather than the submembraneous pH [94]. This may, in part, explain why a study monitoring intracellular pH of *Xenopus* oocytes expressing RhBG via fluorescent dye reported that an ammonium pulse induced an alkalinization of intracellular pH [94], while all studies using microelectrodes to monitor pH_i_ changes Rh-expressing *Xenopus* oocytes detected acidification following an ammonium pulse [87,95–97,101].

## Concluding remarks

Through the development and integration of a wide range of *in vivo* and *in vitro* techniques, our understanding of the extraordinary superfamily of Amt/Mep/Rh has been transformed over the past 30 years. Despite this, the energetics of transport and structural dynamics of the protein during the translocation cycle, remain largely elusive and controversial. This is due, in part, to historical underappreciation of the nuanced physiological differences between individual members of the family. In the pursuit of achieving a ‘complete picture’ understanding of the mechanism of Amt/Mep/Rh, we failed to appreciate that our current understanding is a collage of evidence. In addition, limitations with existing methods may allow subtle features of the proteins to go unnoticed or actively mask them from detection. Thus, it is time to explore new and rapidly developing biophysical and biochemical techniques to solve the fundamental mechanistic questions about Amt/Mep/Rh family. In this context, a standardised approach based on the capture of pure protein from the native lipid environment (e.g., using SMALP) coupled with a finely controlled and adaptable *in vitro* measurement system (such as SSME) could pave the way to the next breakthrough in our understanding of this remarkable superfamily of transporters.

## Open Access

Open access for this article was enabled by the participation of University of Strathclyde in an all-inclusive *Read & Publish* agreement with Portland Press and the Biochemical Society under a transformative agreement with JISC.

## Abbreviations

AMO: ammonia monooxygenase
AOB: Ammonia Oxidizing Bacteria
CCCP: carbonyl cyanide m-chlorophenyl hydrazone
DEPC: diethyl pyrocarbonate
DNP: 2,4-dinitrophenol
FCCP: p-(tri-fluromethoxy)phenyl-hydrazone
HAO: hydroxylamine oxidoreductase
ICL: intracellular loop
MSF: l-methionine sulphone
MSX: L-methionine-DL-sulphoximine
PMF: proton motive force
